# The Genetic Basis of Thermal Reaction Norm Evolution in Lab and Natural Phage Populations

**DOI:** 10.1371/journal.pbio.0040201

**Published:** 2006-06-06

**Authors:** Jennifer L Knies, Rima Izem, Katie L Supler, Joel G Kingsolver, Christina L Burch

**Affiliations:** **1**Department of Biology, University of North Carolina, Chapel Hill, North Carolina, United States of America; **2**Department of Statistics, Harvard University, Cambridge, Massachusetts, United States of America; Duke UniversityUnited States of America

## Abstract

Two major goals of laboratory evolution experiments are to integrate from genotype to phenotype to fitness, and to understand the genetic basis of adaptation in natural populations. Here we demonstrate that both goals are possible by re-examining the outcome of a previous laboratory evolution experiment in which the bacteriophage G4 was adapted to high temperatures. We quantified the evolutionary changes in the thermal reaction norms—the curves that describe the effect of temperature on the growth rate of the phages—and decomposed the changes into modes of biological interest. Our analysis indicated that changes in optimal temperature accounted for almost half of the evolutionary changes in thermal reaction norm shape, and made the largest contribution toward adaptation at high temperatures. Genome sequencing allowed us to associate reaction norm shape changes with particular nucleotide mutations, and several of the identified mutations were found to be polymorphic in natural populations. Growth rate measures of natural phage that differed at a site that contributed substantially to adaptation in the lab indicated that this mutation also underlies thermal reaction norm shape variation in nature. In combination, our results suggest that laboratory evolution experiments may successfully predict the genetic bases of evolutionary responses to temperature in nature. The implications of this work for viral evolution arise from the fact that shifts in the thermal optimum are characterized by tradeoffs in performance between high and low temperatures. Optimum shifts, if characteristic of viral adaptation to novel temperatures, would ensure the success of vaccine development strategies that adapt viruses to low temperatures in an attempt to reduce virulence at higher (body) temperatures.

## Abstract 2

Extensive phenotypic characterisations and detailed genetic analyses of the genome sequences of experimentally evolved phage help to reveal the genetic basis of adaptation to temperature variation in natural populations.

## Introduction

The study of evolutionary responses to temperature has served as an important model for understanding the process of adaptation to novel environments [1], in part because the evolutionary and mechanistic causes of thermal adaptation are relatively transparent. Temperature is a component of the environment that varies predictably, and for which we have knowledge of the proximate mechanisms (e.g. biochemical rate processes) that determine the effects of temperature on growth or performance [2]. Theoretical explorations of thermal adaptation make predictions based on this knowledge [3–5]. For example, trade-offs in performance at different temperatures are expected to result from a biochemical trade-off between enzyme stability and function. Enzymes selected for stability at high temperatures are less functional at lower temperatures, whereas enzymes selected for function at lower temperatures are less stable at high temperatures [6]. However, although performance trade-offs have been observed for individual enzymatic reactions, they have rarely been observed for physiological traits [2]. Most of the studies that did observe trade-offs stand out because they measured performance at five or more temperatures across the entire thermal niche [7–13]. However, few of these studies were sufficiently powerful to describe the nature of the genetic constraint governing the trade-off [8,10].

Thermal adaptation and constraint are viewed most naturally as a type of continuous reaction norm, in which the phenotypic trait value (e.g. fitness or performance) of a genotype varies as a function of some continuous environmental variable (e.g. temperature). Reaction norms are routinely used to investigate environmental effects on a variety of phenotypes. For example, reaction norms have been used to understand how bacterial survival varies as a function of antibiotic concentration [14], immune suppression varies as a function of mold toxin concentration [15], and photosynthesis rate in plants varies as a function of temperature [16].

**Figure 1:**
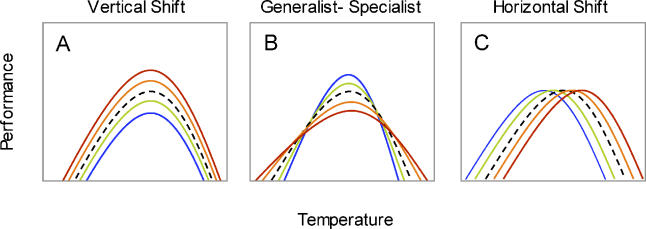
TMV Decomposes Reaction Norm Shape Variation into Three Modes of Variation Vertical shifts produce changes in average performance, horizontal shifts produce changes in the optimal temperature, and generalist-specialist variation produces changes in niche width. An identical template polynomial is shown in each chart as a black dashed reaction norm. The transitions from blue to red curves illustrate hypothetical contributions of the three modes of variation to adaptation to high temperature.

Thermal reaction norms typically have a common general shape in which performance increases with increasing temperature, reaches a maximum at some intermediate temperature, and then declines rapidly with further increases in temperature [17]. Based on knowledge of this common shape, evolutionary physiologists have proposed modes of variation in thermal reaction norms that are of particular biological interest (Figure 1): vertical shift (in average fitness), horizontal shift (in optimal temperature), and generalist-specialist variation (niche width) [18]. A recent study of natural populations of Peiris rapae caterpillars successfully decomposed the quantitative genetic variation in thermal reaction norm shape into these three modes of biological interest, and found that most of the variation was due to generalist-specialist variation [10]. Caterpillar growth rates were either intermediate across a wide range of temperatures, or high at intermediate temperatures and low at extreme temperatures. Studies like this one address the nature of standing variation in thermal reaction norm shape, however, the relative contributions of these different modes to the process of adaptation to novel thermal environments are unknown. For example, is adaptation to high temperatures due primarily to evolutionary changes in average performance, in optimal temperature, in niche width, or in a combination of these modes? How do particular genetic changes selected during evolution contribute to these modes?

Studies of species found naturally in contrasting thermal environments can provide some insight into these questions. For example, comparisons of the effect of temperature on growth rate of actinobacteria strains [19] and the photosynthetic ability of unrelated plant species [16] found in different thermal environments both suggest that adaptation to high temperature occurred primarily via a shift in the optimum temperature. However, these observed patterns are the result of a long history of selection on these species, and do not provide information as to which modes of variation were responsible for the initial adaptation to high temperature (e.g., mutational decay could explain why species no longer perform well at temperatures they no longer experience).

Experiments in which laboratory populations have been adapted to a novel temperature should provide a more powerful means for assessing the contributions of changes in average performance, in optimal temperature, and in niche width to thermal adaptation. Indeed, bacteria and bacteriophage model systems have made considerable progress toward this goal. Investigations of thermal adaptation in Escherichia coli have provided thorough characterizations of phenotypic evolution, measuring thermal reaction norms with sufficient detail that a visual inspection suggests contributions from multiple modes of variation [8,13,20]. However, the relatively large genome of E. coli has made it difficult to associate reaction norm shape changes with particular genetic changes (but see [21]). In contrast, experiments that investigated thermal adaptation in bacteriophage provide detailed analyses of the genetic bases of adaptation, but only a limited characterization of reaction norm evolution [22,23]. Investigations in both model systems failed to analyze thermal performance using continuous reaction norms, thus, limiting their ability to characterize the nature of reaction norm shape changes [10].

To illustrate the power of analyzing evolutionary responses to temperature using continuous reaction norms, we revisit a recent study of high temperature adaptation in the bacteriophages G4 and Φ X174 [22] that stands out because it was able to associate adaptation with particular genetic changes. Holder and Bull demonstrated that phage populations readily evolved higher growth rates at high temperature, but characterized the correlated responses in growth rates only at the ancestral temperature optimum. Here we reexamine the adaptation that occurred in their G4 lineage, measuring performance over a wider temperature range in order to determine how the thermal reaction norm responded to selection for growth at high temperature. We employed a statistical method called Template Mode of Variation (TMV) [10] to decompose the evolution of reaction norm shape into vertical shift, horizontal shift, and generalist-specialist variation. This approach enabled us to quantify the contribution of each mode to adaptation to high temperatures, to identify how specific mutations affect variation in thermal reaction norms, and to identify genetic trade-offs that were not apparent in the earlier analysis. In addition, we investigated whether one of the mutations that had a large effect on reaction norm shape contributes similarly to genetic variation in thermal performance in natural bacteriophage populations.

### Experimental Design

**Figure 2:**
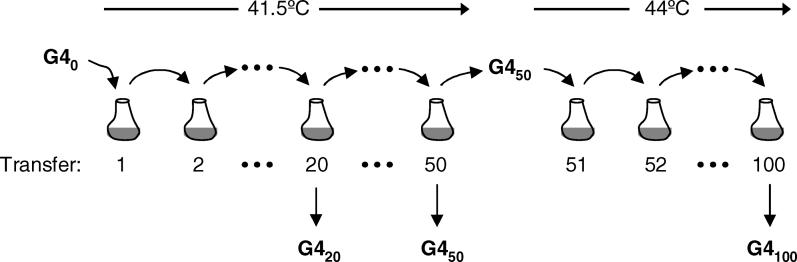
Experimental Design Holder and Bull [22] used a clone of the bacteriophage G4 to found a population that was propagated through 50 serial transfers at 41.5 °C, followed by 50 serial transfers at 44 °C. In each serial transfer 1 × 10^6^ phages were added to a 10 ml culture containing 1 × 10^9^ exponentially growing E. coli hosts, incubated for approximately 45 min at the specified temperature, and then treated with chloroform to kill any remaining hosts. During the 45-min incubation, the phage population size increased to approximately 1 × 10^10^ phages, and 1 × 10^6^ of these phages were used to initiate the next serial transfer. The only exception occurred after the 50^th^ serial transfer, when the population was forced through a single phage bottleneck to homogenize the population. In the current study, we isolated single phage from this evolving population at transfers 20, 50, and 100 (designated G4_20_, G4_50_, and G4_100_). We characterized the performance of these phages and the ancestral G4 (designated G4_0_) across a range of temperatures.

In the evolution experiment conducted by Holder and Bull [22], a single population of the bacteriophage G4 was adapted to the inhibitory temperatures of 41.5 °C and 44 °C and monitored for nucleotide changes throughout its entire 5-kb genome (experimental design is described in detail in Figure 2). Phages were evolved by serial transfer into fresh cultures of rapidly dividing bacterial hosts to select for phage genotypes with the fastest rates of reproduction. Holder and Bull ensured rapid adaptation by selecting for growth beyond G4′s thermal niche boundary, and by maintaining large population size (*N_e_* ≈ 1 × 10^6^), to minimize the effect of genetic drift. Monitoring evolution during an initial 50 serial transfers (approximately 100 generations) at 41.5 °C, and a further 50 serial transfers at 44 °C, Holder and Bull confirmed that adaptation to high temperature occurred rapidly and that it was accompanied by a large number of genetic changes.

In the current study, we isolated five individual phage from this evolving population at transfers 20, 50, and 100. We sequenced the genomes of these phages, and used the isolate most representative of the population consensus at each transfer (as described in Holder and Bull [22]) for all further analyses. Following the convention from Holder and Bull [22], the notation G4*_t_* will be used to represent the phage isolated from serial transfer *t.* Thus, G4_0_ represents the phage genotype used to initiate selection at 41.5 °C, and G4_100_ represents the phage isolated following 100 transfers (50 transfers at 41.5 °C plus 50 at 44 °C). Our characterization of evolutionary responses to temperature in this population expanded on that of Holder and Bull [22] by measuring the growth rates of G4_0_, and the evolved phage, G4_20,_ G4_50,_ and G4_100_, at numerous temperatures that span the thermal niche of G4. Our measure of growth rate is a measure of absolute fitness in the experimental conditions (see Materials and Methods).

## Results

### Responses to Selection at High Temperature

**Figure 3:**
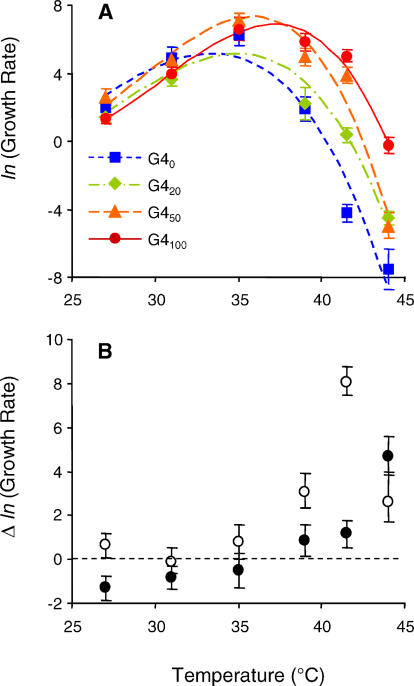
Correlated Responses to Adaptation at High Temperature (A) Reaction norms estimated using TMV explain 71.79% of the sums of squares in the data. Data are mean growth rates ± s.e.m. for each phage genotype determined from three or four replicate measures at each temperature. (B) Correlated responses to selection at 41.5 °C (transfers 1–50; open circles) and to selection at 44 °C (transfers 51–100; closed circles). Data are the differences in mean ln(growth rate) ± s.e.d. between G4_50_ and G4_0_, and between G4_100_ and G4_50_, respectively.

To determine the consequences of adaptation to high temperature, we measured the growth rate of the ancestral phage and three evolved phages at six temperatures between 27 °C and 44 °C (Figure 3). The evolved genotypes were isolated from the Holder and Bull [20] evolving population after the 20^th^, 50^th^, and 100^th^ serial transfers at high temperature, and a visual inspection of the data indicates that evolution occurred between each of these time points (Figure 3A). Reaction norms generated for all genotypes had the overall shape that is characteristic of thermal reaction norms for fitness and performance [18]. Growth rate increased with temperature until a maximum was achieved around 35 °C and declined as temperature increased above this maximum (Figure 3A). Note that either a difference in temperature calibration, or an inadvertent 2-fold difference in salt concentrations produced a difference in phage growth rates between our study and that of Holder and Bull [22]. From our measures, phage did not exhibit positive growth rates at 41.5 °C until transfer 20, or at 44 °C until transfer 100. This calibration difference should not affect the measured shape of thermal reaction norms, it should only affect our ability to pinpoint the exact temperatures to which these phages were adapted.

An examination of the response to selection at 41.5 °C (transfers 0–50) and at 44 °C (transfers 50–100) revealed that evolutionary responses were greater at temperatures above 35 °C than at temperatures below 35 °C (Figure 3B). Correlated responses to selection at both 41.5 °C and 44 °C were positive at temperatures above 35 °C. By contrast, evolutionary responses at temperatures below 35 °C were uncorrelated with the response to selection at 41.5 °C, and negatively correlated with the response to selection at 44 °C (Figure 3B).

### Evolution of Reaction Norm Shape

We analyzed reaction norm shape using TMV [10], a statistical method that assumes each of the measured reaction norms can be described by shifting and stretching a common template reaction norm, represented by a polynomial *P[t]* of temperature *t*. The growth rate measures for all genotypes at all temperatures were simultaneously fit to the model:



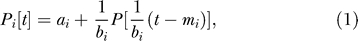



in which both the common template *P[t]* and the parameters *a_i_, m_i_,* and *b_i_* (representing, respectively, the average growth rate, optimal temperature, and niche width for each genotype *i*) are unknowns.

In this model, differences among genotypes in average growth rate *(a_i_)* are modeled by vertical shifts of the template reaction norm (Figure 1A). Average growth rate is the only parameter that does not incorporate a trade-off in performance across temperatures. Differences in optimal temperature are modeled using horizontal shifts that move the entire reaction norm left and right (Figure 1B), positioning the optimum for each genotype at *m_i_* without stretching or skewing the reaction norms. Differences in niche width are modeled by assuming a generalist-specialist tradeoff that constrains the positive area under the growth rate curve *P_i_[t]* to be constant. Thus, increases in niche width yield both a wider temperature range and a lower maximum growth rate (Figure 1C).

**Figure 4:**
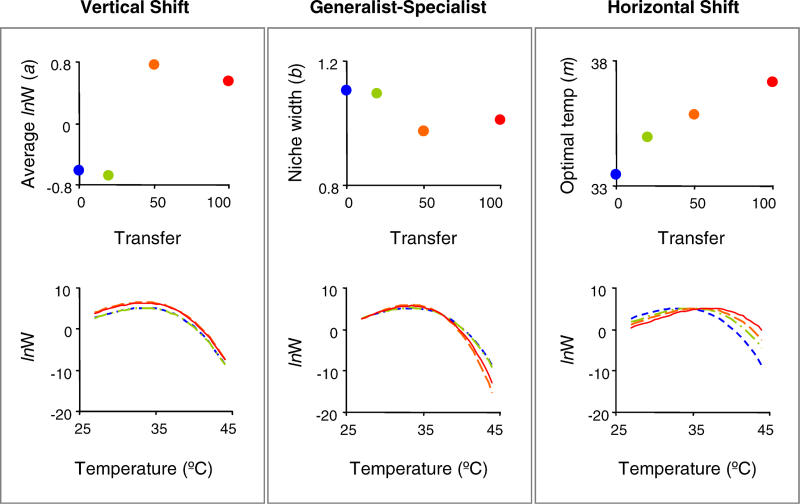
Modes of Variation among the Reaction Norms of Evolving Genotypes TMV was used to decompose the variation among the reaction norms described in Figure 3 into three modes of variation. Each uppermost graph depicts the values of the fitted parameters from the model and the bottom graphs illustrate the impact of each parameter, if considered alone, on the shape of the reaction norm of G4_0._ lnW is the natural log of growth rate.

The model (equation 1) produced by the TMV analysis provides a good fit to the growth rate data at each time point (Figure 3A). The reaction norms conformed to a common shape that was well approximated by a polynomial of degree 3 (see Materials and Methods). The contributions of the three modes to the variation in shape are illustrated in Figure 4. Optimal temperature showed the most consistent pattern of evolutionary change, increasing from 33.4–37.1°C during the experiment, and explained the largest proportion (47.38%) of the shape variation (Table 1). Niche width and average growth rate revealed more complex patterns of change (Figure 4, top), and explained 12.65 and 11.75% of the shape variation, respectively. In total, the modes of variation explained 71.79% of the shape variation, with horizontal shift explaining most of the evolution of reaction norm shape (Table 1).

**Table 1:**
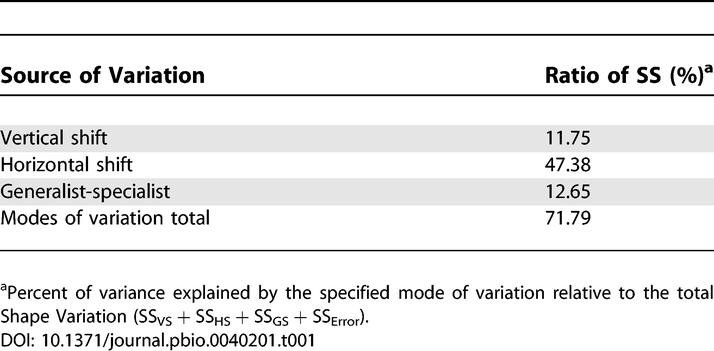
Decomposition of Shape Variation

### Genetic Basis of Reaction Norm Evolution

In order to identify the mutations responsible for changes in reaction norm shape, we sequenced the genomes of the ancestral phage G4_0_ and the evolved phages G4_20_, G4_50_, and G4_100_. Genome sequences indicated that 10 mutations fixed during the 100 serial transfers. Of these, four were present in G4_20_, one additional mutation was present in G4_50_, and the remaining five mutations appeared in G4_100_ (Table 2). Although it was not present in phage from later time points, one additional mutation was present in G4_20_. The location of the ten mutations around the genome showed no significant deviation from a random distribution among the non-overlapping genes (χ^2^ = 3.41; df = 7; *p* > 0.85).

**Table 2:**
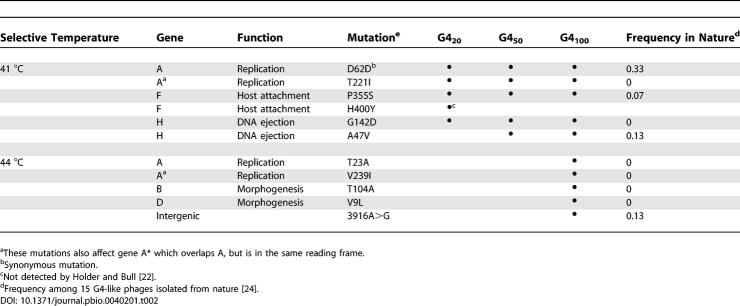
Genetic Basis of Adaptation to High Temperature

**Figure 5:**
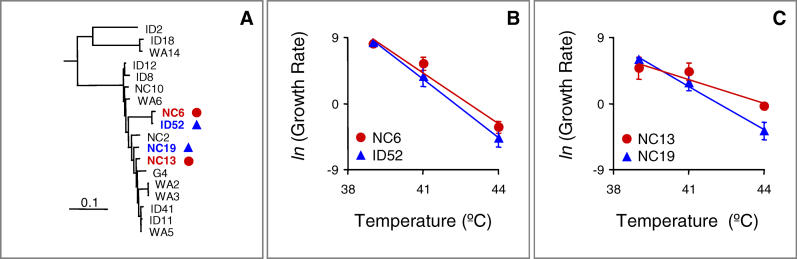
Growth Rates of Phage Isolated from Nature at High Temperatures (A) Phylogenetic relationships of G4-like phages recently isolated from environmental samples, modified from [24]. The genotypes of two pairs of sister taxa that differ at a locus (gene H: A47V) determined to have a large effect on reaction norm shape are indicated with blue (alanine) and red (valine). (B and C) Thermal reaction norms of the two phage pairs. Data are means ± s.e.m. determined from four or five replicate measures at each temperature.

An examination of the genomes of 15 natural isolates of G4-like phage [24] indicated that four of the ten adaptive mutations are polymorphic in natural populations (Table 2). This observation allowed us to explore the possibility that variation at these sites might underlie variation in thermal performance in natural populations (Figure 5). To address this question, we focused on the single mutation present at transfer 50 that was not already present at transfer 20—gene H: A47V. This is the only mutation whose acquisition was unambiguously associated with an increase in growth rate at the selective temperature (41.5 °C). All other increases in growth rate were associated with the acquisition of multiple mutations. We identified two pairs of closely related phage from the natural isolates that differ at this gene H locus (Figure 5A). In each pair, one genotype encodes the mutant amino acid (valine) and the other genotype encodes the ancestral amino acid (alanine). We measured the growth rates of these phages at 39, 41, and 44 °C and found that growth rate depended on temperature, phylogenetic relationship (pair), and genotype at the locus of interest, with significant temperature × pair and temperature × genotype interactions (Table 3). Similar to the outcome of the evolution experiment, the temperature × genotype interaction among natural phage isolates resulted because the A47V mutation was associated with higher growth rates at high temperatures (41 and 44 °C), but not at intermediate temperature (39 °C) (Figure 5).

**Table 3:**
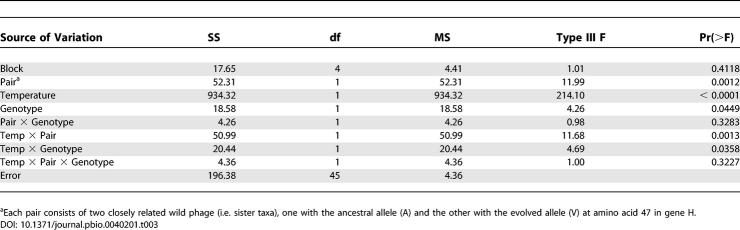
ANOVA Describing the Effect of Gene H Mutation A47V in Natural Phage Isolates

## Discussion

Holder and Bull [22] demonstrated that the phage G4 adapted rapidly to high temperatures (41.5–44 °C) from an ancestral temperature of 37 °C, and identified specific genetic changes associated with adaptation. They also showed that the increased fitness at high temperatures was not correlated with decreased fitness at the ancestral temperature. In our study we considered adaptation of G4 to high temperature in the context of the evolutionary changes in thermal reaction norms over a wide temperature range (27–44 °C). Our measurements indicate that evolutionary increases in fitness (growth rate) at the selected temperatures (41.5 °C and 44 °C) were associated with correlated increases in fitness at temperatures of 39 °C and above, but with no change or decreases in fitness at temperatures of 35 °C and below (Figure 3B). By measuring growth rate over a wider range of temperatures, we were able to detect correlated effects of selection on performance at high temperatures that were not detected by Holder and Bull [22]. However, our data are consistent with the conclusions of Holder and Bull because negative correlations arose over only a small subset of the temperature range, and from only a subset of the genetic changes.

To provide a more detailed picture of the evolutionary changes in thermal sensitivity during adaptation, we applied the TMV statistical method [10] to our data. TMV takes advantage of the continuous nature and common shape of thermal reaction norms to decompose reaction norm evolution into changes in three underlying parameters: average growth rate *(a)*, niche width *(b)*, and optimal temperature *(m)*. Our analysis indicates that changes in average growth rate, niche width and optimal temperature all contributed to the evolutionary changes in thermal reaction norms in this study (Figure 4, top), and that these parameters contributed differently to the evolutionary changes in growth rate at different temperatures (Figure 4, bottom). For example, adaptation (increased growth rate) at higher temperatures was primarily due to evolutionary increases in optimal temperature, with only small contributions from the other two parameters. By contrast, increased growth rates at temperatures near 35 °C were due largely to evolutionary increases in average growth rate.

Both horizontal shifts (optimal temperature) and generalist-specialist tradeoffs (niche width) can generate patterns of positively and negatively correlated changes in performance at different temperatures (e.g. Figure 3B); by using TMV were we able to quantify the relative contributions of these modes of variation to reaction norm evolution [10]. The TMV analysis confirmed that the largest and most consistent evolutionary changes were increases in optimal temperature (Figure 4, top), which accounted for 47.38% of the shape variation in the data (Table 1). In contrast, generalist-specialist variation (niche width) and vertical shifts (average growth rate) contributed only 12.65% and 11.75%, respectively, of the shape variation (Table 1).

It is worth considering whether the contribution of vertical shifts to reaction norm shape evolution in this laboratory population was artificially low because it had been previously adapted to 37 °C, the standard lab condition [22]. Adaptation to the standard lab condition is a common component of microbial evolution experiments, and in this case it was intended to exhaust the adaptive genetic variation that was not temperature specific—i.e. the adaptive genetic variation composed only of vertical shift. Our data are consistent with the expectation that vertical shifts that occur during adaptation to the experimental temperatures should occur only in combination with changes in another mode of variation.

Although the physiological basis of the thermal adaptation is unknown, horizontal shifts are consistent with the idea that increases in protein stability at high temperatures result in decreased protein activity (e.g. enzymatic reaction rates) at lower temperatures [6]. In addition, the random distribution of mutations among genes is suggestive of selection acting for increased protein stability because such selection may be expected to act equally on all proteins. In contrast, selection for particular activities relevant for growth high temperature would likely act with different strengths on different proteins. If this biochemical mechanism does explain most adaptation to novel temperatures, it is surprising that it has been so difficult to detect trade-offs in fitness in animals. It is tempting to think that the simplicity of the virus life cycle underlies the consistency of our finding of horizontal shifts and the expected biochemical underpinnings of thermal performance trade-offs. This is a hopeful perspective because horizontal shifts can be exploited in the development of live cold-adapted vaccines, as in [25]. This vaccine development strategy adapts viral strains for growth at temperatures substantially below human body temperature in an attempt to generate viruses that grow well in cell culture, but are avirulent in humans.

### Investigating Thermal Adaptation and Constraint

Temperature adaptation is generally assumed to be governed by performance trade-offs across temperatures [5,26], despite a shortage of data demonstrating their existence [2]. From the existing data, it is difficult to determine whether trade-offs are in fact rare, or whether the data and analysis methods (usually multivariate analyses of variance in performance at a few temperatures) were not sufficiently powerful to identify trade-offs.

The primary evidence for the latter explanation is that studies that measure performance over wider temperature ranges more often identify trade-offs [7]. Whereas studies that measured performance across most of the thermal niche have identified thermal performance trade-offs in bacteriophage (this study), bacteria [8,11], and insects [7,12], counterparts that measured performance over only a portion of the thermal niche often failed to identify trade-offs (e.g. bacteriophage: [22]; bacteria: [27]; and insects: [28]). Similarly, the power of our analysis derived from the measurement of performance over a wide temperature range. Had we measured performance in this study only at temperatures between 37–44°C, the TMV method would have misleadingly attributed most of the variation in reaction norm shape to vertical shift, and we would have concluded that trade-offs did not influence reaction norm shape variation.

### The Genetic Basis of Adaptation in Laboratory and Natural Populations

Whereas adaptation in the laboratory population described here resulted from selection acting on new mutations, adaptation in nature may often result from adaptive genetic variation that is not new, but already existing in the population. Whether the adaptive mutations identified here also contribute to genetic variation in natural populations will depend in part on whether their effects are neutral (or mildly deleterious) in the natural thermal environment. A comparison of the novel genetic variation generated in lab adaptation to the standing genetic variation present in nature suggests this may be the case. We investigated the polymorphism present in natural populations at sites that contributed to adaptation in the lab by examining a collection of 15 G4-like phage recently isolated from nature [24]. Four of the ten adaptive mutations were polymorphic among these phages. Most of the polymorphisms were rare, present at frequencies of 0.07 or 0.13, as would be expected if these mutations are neutral or slightly deleterious in nature. Furthermore, if we assume that the temperature optimum of ‘lab-naïve' G4-like phage around 29 °C (unpublished data) is indicative of the natural thermal environment, then the minimal effects of the lab adaptive mutations at this temperature (Figure 3, raw data) also suggest their near neutrality in nature.

To examine whether these mutations do contribute to variation in thermal performance in natural populations, we identified one mutation that both contributed conclusively to adaptation in the lab and existed in multiple natural phage isolates. Most of the mutations from the laboratory population of G4 occurred in temporal clusters so we could not identify their individual effects. However, a single adaptive mutation in gene H (A47V) occurred between transfers 20 and 50. This mutation is also present in two of the 15 G4-like natural isolates. Similar to its effects in lab adaptation, the presence of this gene H mutation in the natural isolates is associated with thermal reaction norm shape variation in phages with and without the mutation. In both the experimentally adapted population and among phages isolated from nature, this mutation was associated with higher growth rates at high temperatures, but not at low temperatures. Only one of the other mutations that contributed to lab adaptation was differentially represented among the hot adapted (NC6 and NC13) and the cold adapted (ID52 and NC19) natural isolates. That mutation, gene F: P335S, was found only in phage NC19, which has an ancestral (cold adapted) allele at gene H: A47V. If anything, the fact that this gene F mutation appears in NC19 should have worked against the finding that A47V is associated with adaptation to high temperature in natural phage isolates.

The observation that experimental evolution can result in molecular changes that converge on natural isolates has been made before. Wichman et al. [29] made similar observations during adaptation of the related phages Φ X174 and S13 to alternative hosts. It is possible that these results are unique to phage because of their large population sizes and/or small genomes. However, it is as likely that these observations have been made only in phage systems because, until recently, it has only been possible to identify the genetic basis of lab adaptations in phage [30,31]. The ease with which ours and the previous study identified genetic variations that are associated with phenotypic differences among wild phage suggests that laboratory evolution experiments may often predict the precise genetic basis of adaptations in nature.

## Materials and Methods

### Strains and culture conditions.

G4 populations from the Holder and Bull [22] experiment were obtained from J. Bull (University of Texas, Austin, Texas, United States). Clonal isolates from these populations were designated G4_0,_ G4_20,_ G4_50_, and G4_100._ The G4-like phages, NC6, NC19, ID52, and ID13, were isolated from natural phage populations in 2001 and 2002 [24], and were obtained from H. Wichman (University of Idaho, Moscow, Idaho, United States).

Culture conditions were as in Holder and Bull [22]. Briefly, phage were grown on E. coli C, the standard laboratory host of G4, in LB (5 g Yeast extract, 10 g Bacto tryptone, and 5 g salt/ L) broth supplemented with 2 mM CaCl_2_, and on LB plates (15% agar). LB top agar (0.7% agar) was also supplemented with 2 mM CaCl_2_. Phage were stored for short term (< 1 wk) at 4 °C in this growth media, and for long term at −20 °C in LB broth supplemented with 2 mM CaCl_2_ and 40% glycerol.

### Growth rate assays.

Growth rate assays were designed to closely follow the selection conditions in Holder and Bull [22]. We used 300 μl of a stationary phase E. coli culture to inoculate 10 ml of LB, and incubated this fresh culture in a water bath at the experimental temperature until the culture reached an optical density (OD_600_) of 0.6 ± 0.1, which corresponded to 2–3 × 10^8^ cells/ml at most temperatures (at 44 °C an OD_600_ of 0.6 corresponded to 9 × 10^7^ cells/ml). Approximately 1 × 10^6^ phages were added to this exponentially growing culture, and the mixture was grown for 45 min, at which point a 1-ml sample was treated with 50 μl chloroform to kill bacteria. Phage numbers at the start *(N*_0_ *)* and end *(N*_45_ *)* of the assay were determined by plating. Growth rate was calculated as the increase in phage number over 45 min *(N*_45_/*N*_0_ *)*. Both the experimental serial transfers and the growth rate assays were initiated with a low ratio of phage to bacteria (˜0.001) to minimize the possibilities for competition, and therefore, frequency dependent selection. In this way, we ensure that growth rate is a valid measure of absolute fitness in the experimental conditions. Although the temperatures reported here may have differed slightly from the temperatures reported by Holder and Bull [22], they were measured with a factory-calibrated thermometer that is accurate to within 0.1 °C (Fisher Scientific, Hampton, New Hampshire, United States).

### Analysis of reaction norm shape.

Reaction norm shape was analyzed using TMV [10], a hypothesis driven decomposition of variation in which the principle modes of variation are predetermined by the experimenter. In our case these modes of variation are vertical shift, horizontal shift, and generalist-specialist variation. Because some of these modes are non-linear, the mean curve is not a good representation of the center of the variation in the data. Therefore, TMV estimates a template shape that represents the center of the distribution in the non-linear space of interest.

TMV constrains the template polynomial *P[t]* in three ways to ensure a fit with biological reality. First, the first order polynomial coefficient of the template shape is held at zero to cause the template shape to have a maximum at *t* = 0. This condition makes the *m_i_* parameters identifiable, and interpretable as the location of the maximum of the curve corresponding to genotype *i*. Second, TMV requires that *P[t]* = 0 for two values in the neighborhood of *t* = 0, so that there is a positive finite area under the curve. Finally, TMV requires that *a_i_* = 0, to ensure a unique fit. As a result, the template and all of the curves are constrained to have a positive maximum, and a finite area under the curve in some neighborhood near the maximum. Even with these constraints, the observed data for a genotype could be monotonic within the measured temperature range (the *m_i_* estimated for such a genotype would be outside the measured range). Thus, many possible curve shapes are allowed by the template.

We used TMV to fit the data to a model (eqn 1) in which both the common template polynomial *P[t]* and the parameters *a_i_, m_i_,* and *b_i_* for each genotype are unknowns. In practice, the model was fit by iteratively optimizing the template polynomial *P[t]* and the variation parameters *a_i_, m_i_,* and *b_i_* until the sum of squared errors are minimized. We evaluated the fit of the estimated template polynomial to the data by rescaling the data with respect to the parameter estimates *(a_i_, m_i_, b_i_)* for each genotype, and measuring the residual variation around the template polynomial *P[t]*. We chose to model the common template shape using a polynomial of degree 3 because it was the lowest degree polynomial for which the residuals were random in direction and similar in magnitude across all temperatures, confirming that the polynomial gives a good approximation of the common template shape.

For further details, see a recent study that applied TMV to thermal performance data in natural populations of Pieris rapae caterpillars [10].

### Sequencing.

Genome sequences were obtained from PCR products. Each genome was amplified in two overlapping segments using recombinant *Taq* DNA Polymerase (Invitrogen, Carlsbad, California, United States), purified with ExoSap-It (United States Biological, Swampscott, Massachusetts, United States), and its sequence was determined from 16 overlapping chain-termination sequencing reactions (UNC Genome Analysis Center and UNC Biology ABI 3100- Avant). The primers used for sequencing reactions were as follows, with the nucleotide position indicating the 5′ end, plus or minus indicating which strand (positive or negative) is generated from that primer, and the length of the primer in parentheses; PCR primers: *49+(21), 2702+(21), and 2495+(21), 225+(19);* and sequencing primers: *−49(21), 225(19), 548(19), 937+(21), 1447+(21), 1997+(21), 2495+(21), 2702+(21), 3162+(21), 3645+(19), 4050+(19), 4501+(19), 4945+(19), and 5435+(19).* Sequences were aligned and analyzed in Sequencher (version 4.5, Gene Codes Corporation, Ann Arbor, Michigan, United States). In all cases, the genomic DNA used for PCR was from a single plaque isolate. Up to five isolates were sequenced from the evolved population at each time point. We chose the isolate most representative of the population consensus described in Holder and Bull [22] for use in the present study.

### Characterization of genetic variation for thermal performance in natural phage populations.

We used an ANOVA [32] to assess the effects of phylogenetic relationship (as determined by [24]), genotype at the locus of interest, and temperature on the growth rate of natural phage isolates. Phylogenetic relationship and genotype were treated as class variables and temperature was treated as a quantitative variable. Replicates were collected in blocks (on different days), and block was treated as a random class variable. Residuals were normally distributed (Shapiro-Wilk test: *W* = 0.98, *p* = 0.30) and homogeneous across treatments. The analysis was conducted using the GLM procedure in SAS (version 8, SAS Institute, Cary, NC).

## Abbreviations

TMV: Template Mode of Variation
